# Integrative Analysis of Circulating Metabolite Profiles and Magnetic Resonance Imaging Metrics in Patients with Traumatic Brain Injury

**DOI:** 10.3390/ijms21041395

**Published:** 2020-02-19

**Authors:** Ilias Thomas, Alex M. Dickens, Jussi P. Posti, Mehrbod Mohammadian, Christian Ledig, Riikka S. K. Takala, Tuulia Hyötyläinen, Olli Tenovuo, Matej Orešič

**Affiliations:** 1 School of Medical Sciences, Örebro University, 702 81 Örebro, Sweden; ilias.thomas@oru.se; 2 Turku Bioscience Centre, University of Turku and Åbo Akademi University, FI-20520 Turku, Finland; alex.dickens@utu.fi; 3 Division of Clinical Neurosciences, Departments of Neurosurgery and Rehabilitation and Brain Trauma, Turku University Hospital, FI-20520 Turku, Finland; jussi.posti@utu.fi; 4 Turku Brain Injury Centre, Turku University Hospital, FI-20520 Turku, Finland; mehmoh@utu.fi (M.M.); riikka.takala@gmail.com (R.S.K.T.); Olli.Tenovuo@tyks.fi (O.T.); 5 Department of Clinical Neurosciences, University of Turku, FI-20520 Turku, Finland; 6 Department of Computing, Imperial College London, London SW7 2AZ, UK; ledig.christian@gmail.com; 7 Perioperative Services, Intensive Care Medicine and Pain Management, Turku University Hospital and University of Turku, FI-20520 Turku, Finland; 8 Department of Chemistry, Örebro University, 702 81 Örebro, Sweden; tuulia.hyotylainen@oru.se

**Keywords:** blood biomarkers, magnetic resonance imaging, mass spectrometry, metabolomics, traumatic brain injury

## Abstract

Recent evidence suggests that patients with traumatic brain injuries (TBIs) have a distinct circulating metabolic profile. However, it is unclear if this metabolomic profile corresponds to changes in brain morphology as observed by magnetic resonance imaging (MRI). The aim of this study was to explore how circulating serum metabolites, following TBI, relate to structural MRI (sMRI) findings. Serum samples were collected upon admission to the emergency department from patients suffering from acute TBI and metabolites were measured using mass spectrometry-based metabolomics. Most of these patients sustained a mild TBI. In the same patients, sMRIs were taken and volumetric data were extracted (138 metrics). From a pool of 203 eligible screened patients, 96 met the inclusion criteria for this study. Metabolites were summarized as eight clusters and sMRI data were reduced to 15 independent components (ICs). Partial correlation analysis showed that four metabolite clusters had significant associations with specific ICs, reflecting both the grey and white matter brain injury. Multiple machine learning approaches were then applied in order to investigate if circulating metabolites could distinguish between positive and negative sMRI findings. A logistic regression model was developed, comprised of two metabolic predictors (erythronic acid and *myo*-inositol), which, together with neurofilament light polypeptide (NF-L), discriminated positive and negative sMRI findings with an area under the curve of the receiver-operating characteristic of 0.85 (specificity = 0.89, sensitivity = 0.65). The results of this study show that metabolomic analysis of blood samples upon admission, either alone or in combination with protein biomarkers, can provide valuable information about the impact of TBI on brain structural changes.

## 1. Introduction

Traumatic brain injury (TBI) is a major cause of death and disability worldwide [1,2], particularly in children and young adults. TBI impairs affected individuals’ ability to operate at a functional level as well as puts a great burden on healthcare systems [3]. The standard evaluation of the severity of the TBI is based on the Glasgow Coma Scale (GCS) upon admission, which is used as a tool to assess the required level of the treatment intervention [4], or to determine appropriate diagnostic tests (e.g., if a head CT-scan is required for further evaluation). Based on the GCS evaluation, TBI can be classified into three severity groups: mild TBI (mTBI), moderate TBI (moTBI), and severe TBI (sTBI), which are, however, still crude predictors of outcome [5]. TBI commonly leads to metabolic disruptions, including energy crisis and/or failure [6,7]. It is imperative, even in the early stages following TBI, to determine the severity of the injury in greater detail, the long-term recovery options, and work towards personalized treatment to increase the chances of full recovery.

One way to move towards personalized treatment is through the discovery of biomarkers sensitive to the pathophysiological processes in TBI. Previous studies have shown that S100 calcium-binding protein B (S100B) [8], branch-chained amino acids [9], brain glucose levels [10], ubiquitin carboxy-terminal hydrolase-L1 (UCH-L1) [11,12], as well as glial fibrillary acidic protein (GFAP) [13,14], tau protein [15], heart fatty-acid binding protein (H-FABP), and neurofilament light polypeptide (NF-L) [16], can be useful biomarkers when assessing the severity of TBI and detecting intracranial gross pathologies. In a recent systematic review of TBI biomarkers, the authors argued that the only biomarker currently reliable enough for the clinical use is S100b [15]. However, S100B has been found to be associated also with extracranial trauma and even with high exercise levels [8].

Previously, we found that circulating small molecules (metabolites) are a potentially-rich source of TBI biomarkers [17], a finding which is supported by earlier studies focusing on amino acids [9]. Circulating metabolites can also help determine those TBI patients needing computed tomography (CT) scans after initial [18]. Previous studies have also evaluated the correlation of blood protein biomarkers and biomarker panels to CT findings of TBI [16]. Although CT scanning is the standard imaging technique in TBI, patients with negative CT scans can have positive structural magnetic resonance imaging (sMRI) findings, while GFAP concentrations within 24h of injury are potentially a good predictor for determining which patients with negative CT findings may require sMRI scans [19]. Furthermore, GFAP was previously found to be an adequate predictor for determination of both CT and MRI positive/negative scans [20].

The link between circulating metabolites and sMRI findings has not yet been investigated. Here we studied the association between circulating metabolites and acute MRI findings in a clinical cohort study comprised of patients with varying degrees of TBI severity. The study included 96 patients from whom serum metabolomics and sMRI data were taken at around three weeks post-injury, and for whom CT results were available. We also explored if it was possible to predict the presence or absence of visible MRI abnormalities, using a combination of metabolites and protein biomarkers from serum samples, taken upon admission to the emergency department.

## 2. Results

### 2.1. Study Setting and Data Survey

**Figure 1:**
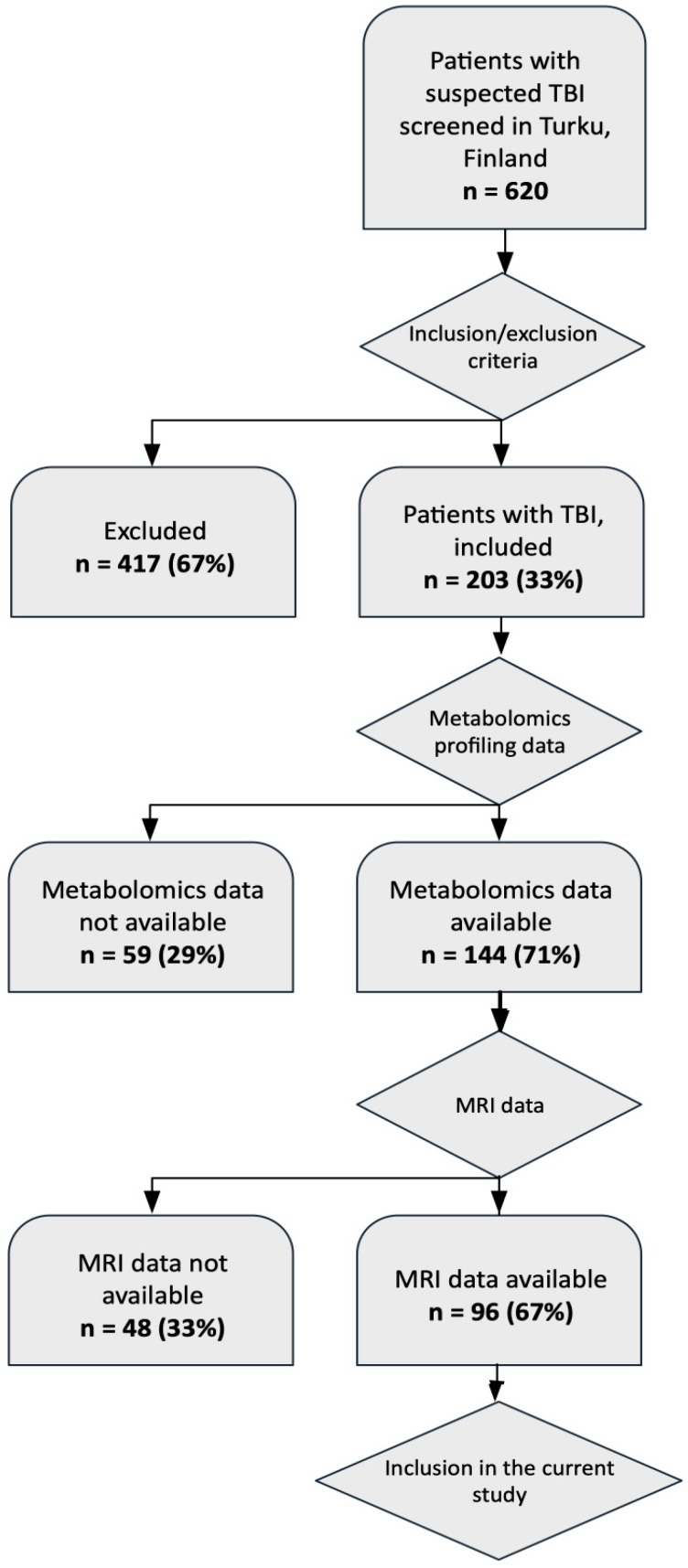
Flow chart showing how the patients were selected for the study.

The study included 96 patients with TBI with available metabolomics and MRI data (Table 1). Inclusion was as shown in the flow chart (Figure 1) and described in the Methods section. For these patients, 451 serum metabolites were measured by using two-dimensional gas chromatography coupled to time-of-flight mass spectrometry (GC×GC-TOFMS), as described previously [17]. In the same patients, sMRI images were acquired and volumetric data were extracted at the acute stage of disease (138 metrics), as previously described [21]. The patients were classified, according to the lowest pre-hospital GCS, into three groups, mTBI (n = 79), moTBI (n = 10), and sTBI (n = 7).

Initially, in order to survey the data as a whole, the metabolites were summarized by K-Means clustering [22], while the sMRI image data were decomposed using Independent Component Analysis (ICA) [23]. Circulating metabolites were grouped into eight distinct clusters, which are summarized in Table 2. The sMRI image data were decomposed into 15 independent components (ICs). This technique allows brain volumes which change by similar patterns across individuals to be grouped together.

**Figure 2:**
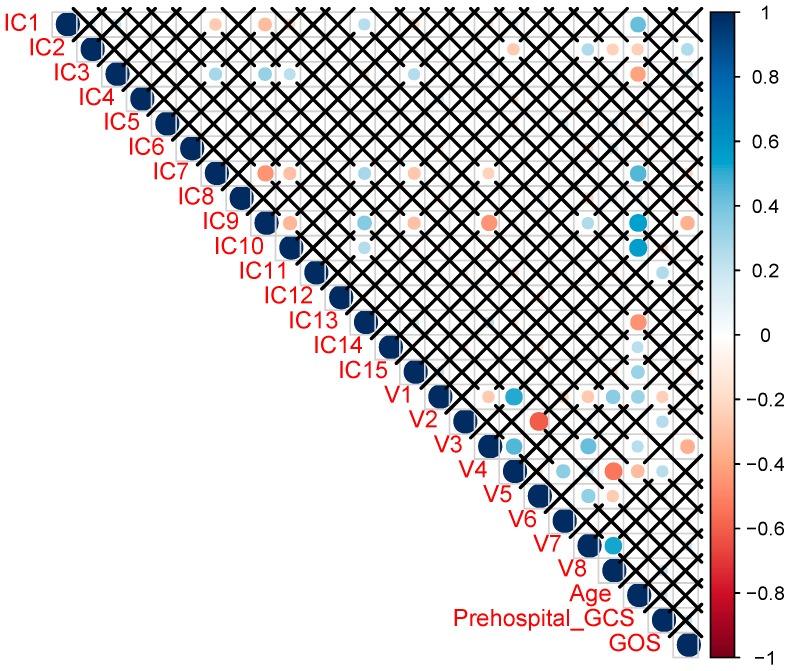
Partial correlations of independent components (ICs) derived from sMRI data with the eight metabolite clusters (Vs), as well as age, GCS, and GOS. Partial correlations among all pairs are shown on that matrix, and when a pair is shown as X, the correlation is not significant (*p*-value > 0.05). For the pairs with significant correlations, a bullet is plotted, with its size, transparency and color corresponding to the correlation value (blue signifies positive correlation, red negative).

**Figure 3:**
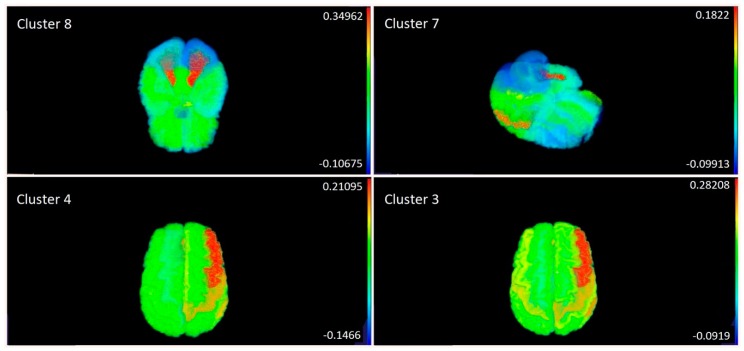
Correlations between four specific metabolite clusters (see Table 2) and sMRI data from different brain areas (on the scale to the left, from bottom to top: minimum to maximum).

Partial correlations between metabolite clusters and ICs are shown in Figure 2. On that trigonal correlation matrix, we see, as variables, 15 ICs (numbered IC1-IC15), eight metabolite clusters (numbered V1-V8), age, pre-hospital GCS and GOS. Metabolite clusters 1, 2, 5, and 6 showed no correlation to the ICs, while clusters 3, 4, 7, and 8 correlated with at least one of the ICs. For the four clusters that showed correlation with the sMRI data, correlations with specific areas of the brain were calculated and projected onto a manually-segmented reference brain, that was used to generate a probabilistic brain atlas from the OASIS database [24]. Strong correlations of these four metabolite clusters with distinct areas of the brain were observed (Figure 3). The top brain regions that correlated positively with Cluster 3 were the right anterior cingulate gyrus, right anterior insula, right middle frontal gyrus, left planum polare, and the right supramarginal gyrus. The top brain regions which correlated positively with Cluster 4 were the right caudate, left lateral ventricle, right lateral orbital gyrus, right middle frontal gyrus, and the left middle occipital gyrus. The top brain regions that correlated positively with Cluster 7 were the right fusiform gyrus, right middle occipital gyrus, left planum polare, and the left superior temporal gyrus. The top brain regions that correlated positively with Cluster 8 were the right cerebellum white matter, left cerebellum white matter, right cerebral white matter, left cerebral white matter, and the left gyrus rectus.

### 2.2. Metabolites Associate with Positive sMRI Findings

**Figure 4:**
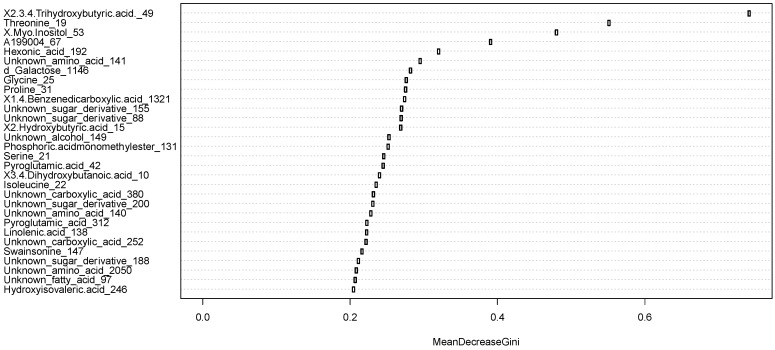
The metabolites that correlate the most to the MRI findings according to mean decrease of Gini index according to the random forest model.

In order to see if the metabolites were also able to predict the need for a MRI scan, we evaluated associations of metabolites with visible sMRI positive/negative findings using a random forest model [25,26]. The metabolites were sorted based on their influence on the mean Gini decrease index [27]. Higher values of mean Gini decrease index indicate higher variable importance, when predicting a target variable. It is virtually a weighted average of the importance of the specific variable across all decision tress that make up the random forest. In total, 31 metabolites were selected (MeanDecreaseGini > 0.2) (Figure 4). The more important a variable, the higher it is ranked in Figure 4. Furthermore, the Welch *t*-test found 34 metabolites which had significantly different values (*p* < 0.05) between the positive/negative sMRI groups. Twelve metabolites overlapped between the random forest model and those from the Welch *t*-test (*p* < 0.05) (Table 3). However, following correction for False Discovery Rate (FDR), there was only one metabolite with a significant difference between the two MRI groups, this being (2,3,4-trihydroxybutyric acid, also known as erythronic acid), which is also the metabolite with the highest importance in the random forest model (Figure 4).

### 2.3. Discrimination of Positive vs. Negative sMRI Findings with Circulating Metabolites

The predictive ability to discriminate positive vs. negative findings from sMRI with circulating metabolites was also evaluated by two support vector machine (SVM) models and by logistic regression. In the first SVM model, the metabolites that were selected as predictors were the ones that belonged in both groups (random forest and Welch *t*-test) (Table 3). In the second SVM model, only erythronic acid was used as a predictor. For the logistic regression model, all 12 features from Table 3 were used as predictors and only the significant ones were kept for the final model.

**Figure 5:**
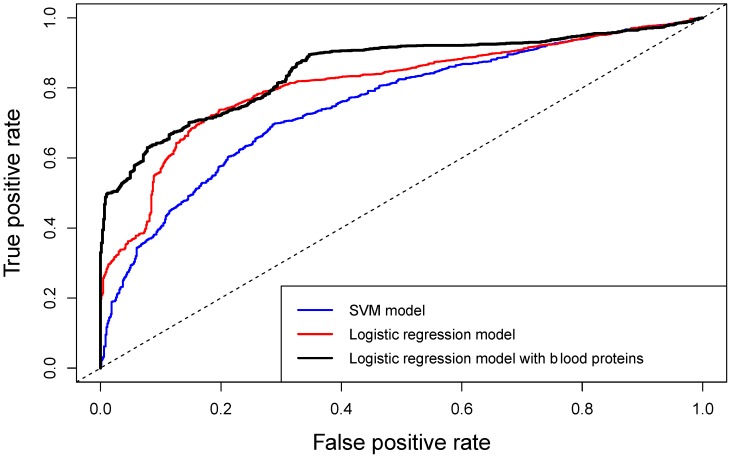
Average ROC curves for three different models, discriminating the positive vs. negative sMRI findings: (blue) Support vector Machine (SVM) model with metabolites, (red) logistic regression model with metabolites, and (black) logistic regression model with metabolites and blood proteins.

For the SVM model with all 12 predictors (100 model runs), mean accuracy of predictions was 0.75, sensitivity 0.78, and specificity 0.54 to discriminate patients with sMRI positive and negative findings. The area under the curve (AUC) of the receiver-operating characteristic (ROC) curve was 0.75 (95% CI: 0.73–0.77) (Figure 5). The model with a single predictor had AUC of 0.61, sensitivity of 0.74, and specificity of 0.33. In the logistic regression model, initial 12 metabolites were reduced to two (erythronic acid and *myo*-inositol), because they were the only two identified as significant by the model. The mean results from the 100 logistic regression models had AUC of 0.81 (95% CI: 0.79–0.82), accuracy 0.76, sensitivity 0.85 and specificity 0.51 (Figure 5).

Since the more severe cases of TBI will most likely have MRI findings, we also examined if the more severe cases are driving the metabolomics findings. The feature selection process and the logistic regression model was run again, but only by including patients with mTBI. The model improved slightly, with an AUC of 0.83 (95% CI: 0.82–0.85), sensitivity of 0.85, and specificity of 0.58. The two key metabolites behind this model were again erythronic acid and *myo*-inositol, with a third significant metabolite being xylobiose. However, this last metabolite did not add considerably to the model, because its exclusion from the model resulted in a model with an AUC of 0.82.

### 2.4. Discrimination of Positive vs. Negative sMRI Findings with Circulating Metabolites Together with Protein Biomarkers

Next, we examined if adding protein biomarkers to circulating metabolites improved the models for discriminating positive and negative sMRI findings. For the feature selection of this extended model, protein biomarkers that were available from the same cohort at admission (GFAP, S100B, UCH-L1, H-FABP, IL-10, total tau (t-tau), and NF-L) were added to the 12 metabolites from Table 3. The random forest model with the 18 predictors (12 metabolites and 6 proteins) showed that the most important protein biomarkers were NF-L, GFAP, H-FABP, and UCH-L1. However, the Welch *t*-test with FDR correction showed that only GFAP, H-FABP, and UCH-L1 had significantly different values between the two sMRI groups. These three blood proteins were added to the SVM model together with erythronic acid to evaluate the improvement of the model in discriminating the two MRI groups. The AUC improved to 0.78, however, sensitivity and accuracy were reduced (0.50, 0.71). For the logistic regression model, the proteins were first assessed, and only NF-L was found to be significant by the model. The logistic regression model had therefore three predictors, erythronic acid, *myo*-inositol, and NF-L. The results from this analysis showed great improvement of the predictive performance of the model, with AUC increased to 0.85 (95% CI: 0.84–0.87), accuracy to 0.82, specificity to 0.89 and sensitivity to 0.65 (Figure 5).

The logistic regression model with the three predictors, erythronic acid, *myo*-inositol, and NF-L, was also evaluated on a subset of the patients who had negative CT scan findings. The results from this analysis showed that the model can facilitate determination of which patients with negative CT scans might require MRI, with AUC of 0.71, accuracy 0.71, sensitivity 0.70 and specificity 0.73, which is in line with the results presented previously [20].

## 3. Discussion

Here we have shown that panels of circulating metabolites, alone or in combination with protein biomarker levels previously reported to be associated with TBI severity, can be good predictors of positive vs. negative sMRI findings in TBI patients. Moreover, our findings show that a panel of metabolites can also facilitate in distinguishing which patients with negative CT findings might have positive sMRI findings.

Identification of inexpensive and non-invasive biomarkers is currently an unmet medical need in TBI [28]. Circulating metabolites hold promise in this area [29]. It has, for example, already been shown in human studies that metabolites associate with severity of TBI and can serve as predictors of patient outcomes [17] as well as help in the identification of patients who need a CT scan [18].

Survey of the complete metabolome and sMRI data, following dimensionality reduction, indicated that four metabolite clusters had significant associations with specific brain regions (Figure 3). The idea that there could be specific metabolic signatures associated with brain injury has been shown previously in an animal model of neurodegeneration where the polar metabolites of different brain regions were measured from multiple brain regions. These metabolic patterns changed depending on the brain region following excitotoxic neuronal injury [30]. Metabolic Cluster 8 correlated primarily to supra- and infratentorial white matter areas suggesting that the metabolites in this cluster could be a signature of white matter injury. The metabolites in this cluster were mainly amino acids (Table 3). All the other metabolite clusters (3, 4, and 7) predominantly associated with changes to cortical grey matter regions. This suggests that the damage to the cortical regions can lead to specific metabolite patterns in the serum. Cluster 7 also correlated with a brain region closer to the brain stem; the right fusiform gyrus (Figure 3). This metabolite cluster is of interest as it contains erythronic acid, which was also indicative of positive sMRI findings. This deeper brain region is likely to be damaged due to shearing forces following the injury [31,32]. Although this was not a focus of the present study, our data does suggest that metabolites may also be useful in determining the type of brain injury. Further functional studies are clearly merited, linking the circulating metabolites and brain metabolome with structural and functional brain changes in TBI.

The findings of this study show that erythronic acid and *myo*-inositol can, on their own, produce models with good discriminatory ability. Of these two metabolites, *myo-*inositol, which is a highly-abundant metabolite in the brain, has been shown to increase in the brain following an injury, both in children and adults [33,34]. The exact role of *myo*-inositol in the CNS remains unclear, however, it has been suggested that it could be an indication of changes in glial cells [35] or changes in osmolarity [36]. In our data, *myo*-inositol was found to be elevated in severe TBI (fold change (FC) vs. healthy controls = 1.36), with the same trend observed in moderate TBI (*p* = 0.1; FC = 1.2) [17]. Erythronic acid is less established as a brain injury marker. It has been suggested that changes in this metabolite may reflect dysregulation of pentose–phosphate pathway [37]. Given that increased anerobic resperation has been observed following a TBI, this could explain the alterations in the pentose phosphate pathway, which is an alternative pathway for anerobic glycolysis [38]. Interestingly, in our data, erythronic acid was found elevated in mild TBI (FC = 1.75), but not in moderate and severe TBI [17].

The addition of NF-L considerably improved the predictions of the positive/negative sMRI findings model. Our findings also suggest that S100b and GFAP are not as prominent protein biomarkers as NF-L in our samples, when predicting positive vs. negative sMRI findings, because they were not as important to the models. This could be down to the relatively short half-lives of these proteins and the delay in sampling of our subjects [11]. This is, however, in line with recent findings, where NF-L was identified as a prominent protein biomarker for discriminating between positive and negative CT scans [16]. Among the metabolites, erythronic acid was a major metabolite capable of discriminating between negative and positive sMRI findings. Erythronic acid is a sugar derivative which is commonly found in aqueous humor of the eye [39] and in the cerebrospinal fluid [40]. It is derived either from the degradation of ascorbic acid or from glycated proteins. The fact that erythronic acid is a metabolite enriched in the central nervous system (CNS), suggests that the observed differences may reflect metabolic changes in the brain, and, possibly, changes in the permeability of the blood brain barrier.

Typical comprehensive untargeted metabolomics assays, such as used in the present study, cover hundreds of metabolites. Most of the metabolites are measured semi-quantitatively (relative concentrations, with respect to set internal standards). Cost and complexity of such analysis as well as lack of accurate quantification for most metabolites, together make such an analytical approach challenging, if not prohibitive, for use in the clinic. However, once metabolites of interest are known, once can develop a fast, targeted assay, which is amenable to use in a typical clinical chemistry laboratory, for a fraction of the cost of comprehensive metabolomics analysis. We have, for example, recently developed just such a targeted metabolomics platform for use in the diabetes clinic [41]. One can thus foresee that, once more evidence on the diagnostic and prognostioc value of specific metabolites in TBI is assembled, a similar TBI metabolite test could be developed for clinical use.

## 4. Limitations

A major limitation of this work is the difference in times of blood sample acquisition (upon patient arrival) and the time of MRI scanning. The range of the days when MRI scan was taken after the injury was 1–50 days, which may have impacted the results, particularly as related to the associations of blood metabolites with sMRI data (Figure 3). However, based on a recent study, where MRI scans were taken at three time points (> 72h, at 3 months and at 12 months after the injury), the authors found that in mTBI (of note, in our study 79 out of 96 patients had mTBI), clinical MRI examinations can be reliably performed at a later time point [42]. Moreover, the time of the MRI was controlled for in the analysis.

Especially for the protein measurements, the arrival blood sample was not drawn at a fixed time post-injury, which can lead to variations in both protein and metabolic markers and in their diagnostic capacity. However, our previous metabolic study demonstrated that the majority of the metabolites were indeed stable over the first few days [17]. This has not been shown to be the case for protein biomarkers, where very careful attention needs to be paid to the time after injury. For example, it is possible to generate a temporal model for s100B, which demonstrates how much this protein biomarker changes in the first few hours following the injury [43]. Given the small sample size, it was also not possible to perform independent validation of the predictive models discriminating positive and negative MRI findings. This will be the focus of the future studies. Nevertheless, here we have performed internal cross-validation with resampling and generation of 100 models for each method, by using each time 70% of the sample for model training and 30% of the sample for model testing.

A relatively small number of patients who visited the Emergency Department during the recruitment are included in this study. We have previously published a study examining the representativeness of the cohort. In short, altogether 620 patients with head injury admitted to the Emergency Department were identified by research assistants. The initially-included patients differed from all admitted patients only in terms of gender distribution [44].

As this is a preliminary study, we intended to study only gross pathologies and investigated simple structural abnormalities, e.g., hemorrhages. Utilizing advanced techniques such as diffusion tensor imaging could improve injury classification due to their capability of detecting subtle structural abnormalities especially in patients with mild injury [45].

The study cohort can be regarded slightly heterogeneous as the majority of the patients sustained an mTBI and only a small portion an sTBI. However, this can also be considered as a strength of the study, as biomarker levels remain lower in patients with mild injury, which usually results in less significant findings diagnostic accuracy in biomarker studies [16,17]. Furthermore, patients with mTBI in this cohort had relatively serious intracranial abnormalities due to a selection bias as mTBI patients with minor symptoms were discharged and those in need of a hospital follow-up were recruited. Lastly, the relatively small study cohort increases the risk of over-fitting bias.

## 5. Materials and Methods

### 5.1. Ethics Statement

This prospective study was part of the collaborative project TBIcare (Evidence-based Diagnostic and Treatment Planning Solution for Traumatic Brain Injuries), funded by the EU 7^th^ Framework Programme (grant agreement number 270259), where we recruited patients with acute TBIs at the Turku University Hospital, Finland, during November 2011–October 2013. The South-West Finland Hospital District Research Ethics Committee approved the protocol (decision 68/180/2011; June 16, 2011). All patients or their next of kin were given both oral and written information about the study and a written informed consent was obtained. All patients were treated according to standard local guidelines based on the actual international guidelines and recommendations [46].

### 5.2. Data Description

During the recruitment process, 620 patients with acute head injury were admitted to the Emergency Department of Turku University Hospital, Finland. Among these, 203 patients were both screened and considered eligible as they met the following inclusion criteria: (i) age ≥18 years, (ii) clinical diagnosis of TBI and indications for acute head CT according to the NICE (National Institute for Health and Care Excellence) criteria [47], and did not meet the following exclusion criteria: (i) blast-induced or penetrating injury, (ii) chronic subdural hematoma, (iii) inability to live independently due to a pre-existing brain disease, (iv) TBI or suspected TBI not needing head CT, (v) more than two weeks from the injury, (vi) not living in the hospital district thereby preventing follow-up visits, (vii) not speaking native language (Finnish), or (viii) no consent received. The current dataset consists of a subsample of the data discussed previously [17], from a patient cohort in Turku, Finland. In that study, blood samples from 144 patients (Turku cohort) were collected within 12 h upon admission. For the current study, we utilized and additional exclusion criterion: (ix) metabolomics and sMRI data not available.

The blood samples were analyzed and the concentration of metabolites on the blood stream was calculated. Early MRI data was available for 96 of the 144 patients. There are 96 patients for which both the metabolomics analysis results and sMRI data (including findings) are available. The sMRI findings were classified into 10 categories (Table 4).

Twenty-six patients had normal findings (0-no injury), 67 patients had at least 1 code of categories 1–9, and for three patients the codes were missing. For the remaining patients two groups were formed: negative MRI (26 patients) and positive MRI (67 patients), which included all patients with at least one abnormal finding. All patients had undergone a routine CT scan at the hospital and 26 patients that had CT negative results had MRI positive results. The full coding and the demographics of the patients can be found in Table 1.

The large majority of patients (82%) were suffering from mTBI (GCS 13–15), with only a few cases of sTBI (7%) (GSC 3–8). This is reflected on the mean pre-hospital GCS (13.5). The sMRIs were taken on average 19.5 days after the injury (median: 17, standard deviation: 13.9).

### 5.3. Metabolomics Analysis

Metabolomic analyses of serum were performed at VTT Technical Research Centre of Finland (Espoo, Finland) as described earlier [17]. Additional analyses to identify metabolites of interest were also performed at LECO Corporation (St. Joseph, Michigan) and at Steno Diabetes Center (Gentofte, Denmark).

In brief, serum samples (30 μL) were spiked with 10 μL of internal standard mixture (C17:0, deuterated valine and succinic acid-d4) and: 400 μL of methanol was added to precipitate the proteins. After evaporation, two step derivatization with methoxyamine hydrochloride and N-methyl-N-trimethylsilyltrifluoroacetamide was done, a retention index standard mixture (n-alkanes) and an injection standard (4,4′-dibromooctafluorobiphenyl), were added and the samples were analyzed by GC×GC–TOFMS consisting of an Agilent 6890 gas chromatograph equipped with a split/splitless injector (Agilent Technologies, Santa Clara, CA, USA), cryogenic dual-stage modulator and time-of-flight mass spectrometer (Leco Corp., St. Joseph, MI, USA). In addition, multipurpose sampler with Maestro software (Gerstel, Mülheim an der Ruhr, Germany) was used for derivatization and sample introduction. Data pre-processing was performed using a combination of the ChromaTOF software and in-house developed software Guineu [48] and the identification was done with NIST 2008 Mass Spectral Library (National Institute of Standards and Technology, Gaithersburg, MD, USA), in-house spectral library and Golm metabolome database (Max Planck Institute of Molecular Plant Physiology, Potsdam-Golm, Germany) [49]. Only the metabolites detected in over 70% of the samples in each of the four study groups were included in the dataset. This procedure resulted in a total of 465 metabolites.

The literature values were obtained from NIST 2008 Mass Spectral Library or they were determined experimentally with GC×GC-TOFMS instrument in our laboratory with authentic standards (in-house developed library). In addition, Golm metabolome database [49] was used for further identification of the metabolites. In cases where the metabolite identity could not be determined with available methods, the chemical class was reported based on the MS spectra [48]. The orders of both sample preparation and analysis were randomized, and a set of controls samples (pooled serum samples), standards and blank samples (solvent blank) was analyzed together with the samples. In the discovery set, the day-to-day variation of internal standards added to all samples was on average 17.3 % and the day-to-day variation in control serum samples (n = 31) of the quantified metabolites was 18.0 %. In the validation set, the variation of internal standards added to all samples was on average 12.3 % and the variation of control serum samples (n = 14) of the quantified metabolites was 9.2 %. That analysis resulted in a total number of 455 metabolites.

Four metabolites had identical values for all patients and were removed from subsequent analysis. A total of 131 metabolites were identified, while the rest were unknown. The metabolites were first log-transformed and then normalized. Apart from the circulating metabolites, the concentration of the blood proteins that were highlighted in the introduction, namely: GFAP, S100B, UCH-L1, H-FABP, IL-10, t-tau, and NF-L, whose quantification has been described previously [16], were also calculated and included in the subsequent analysis.

### 5.4. MRI Analysis

T1-weighted magnetization-prepared rapid gradient-echo (MPRAGE) MR images were obtained from 96 patients using a Magnetom Verio 3T MRI scanner (Siemens Healthcare, Erlangen, Germany), with the following parameters: TE 2.98 ms, TR 2300 ms, flip angle 9°, isotropic voxel size of 1mm × 1mm × 1mm, and matrix size of 240 × 256 × 176. Additionally, T2-weighted, fluid-attenuated inversion recovery, susceptibility weighted, and diffusion-weighted MR images were acquired. Structural MR images were further investigated by experienced neruoradiologists and injury classification was done based on MRI findings. The volumes of interest were generated from the T1-weighted MR images by using the multi-atlas label propagation with expectation-maximization (MALP-EM) pipeline described previously [21]. For the sMRI data, the 138 features that were created were processed though principal component analysis (PCA) and ICA. PCA was applied to evaluate the number of combined features necessary to reduce the dimensionality of the dataset while maintaining enough of the original information. As a threshold for this study, we set that 75% of the original information is enough, since retaining 90% would be to many features compared to the number of observations available (34 principal component account for 90% of the variation when 15 components account for 75% of the information). For that reason, we decided that 15 ICs would be extracted. The *fastICA* package in R statistical software *v*. 3.6.1 (R Foundation for Statistical Computing, Vienna, Austria) [50] was used to perform ICA.

### 5.5. Data Analysis

#### 5.5.1. Clustering of Metabolites

**Figure 6:**
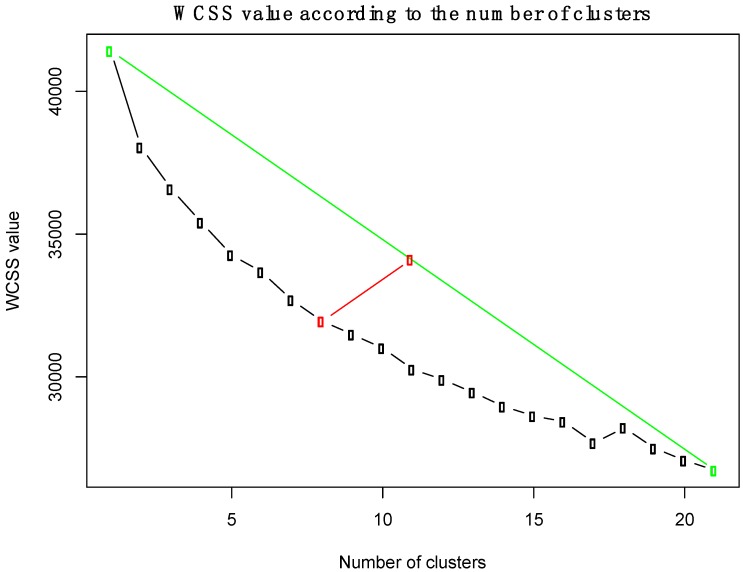
Within-cluster sum of squares (WCSS) errors for the K-means clustering algorithm, according to the number of clusters. The green line connects the first and last point of the WCSS curve. The red line is the “elbow” point, which is the maximum distance between the WCSS curve and the green line.

The 451 metabolites were summarized into clusters. A k-means method was selected as, when compared to other clustering methods (self-organizing maps, hierarchical clustering), it provided a more balanced cluster distribution. The number of clusters was selected based on minimizing the within-cluster sum of squares (WCSS), while maximizing the lines that connects the minimum and maximum values of the WCSS for the number of clusters explored (Figure 6). Based on these results, the number of clusters was shown to be eight. The mean value of the metabolites belonging to each cluster was calculated and these values were used for further analysis. Therefore, the data from 451 metabolites were reduced to eight cluster variables.

#### 5.5.2. Feature Selection

Feature selection, or dimensionality reduction, refers to the selection of a subset of features that could be useful for predictive modeling, either in classification or regression analysis settings. Two different methods were explored for feature selection. First, a Welch *t*-test was performed to compare the mean values of the metabolites in the two MRI groups (positive vs. negative findings) for significant differences. False discovery rate (FDR) [51] correction was applied to adjust for multiple testing. Then, a random forest model which returns each variable’s importance for a specific task, based on the accuracy improvement the feature adds to the model. The R package *randomForest* was used for this task and the package *onewaytests* was used for the Welch *t*-test.

#### 5.5.3. Correlation Analysis—Mapping

The final dataset contained eight metabolic clusters and 15 MRI features for the 96 patients. Partial correlation analysis between the MRI features and the eight clusters was performed. The goal of this analysis was to find which clusters correlate to the MRI features, and then investigate with which areas of the brain these clusters correlate to. For the partial correlation analysis, the R-package *ppcor* was used and for the visualization the Carimas software (Turku PET Center, Turku, Finland) was used.

#### 5.5.4. Predictive Analysis

The goal of the predictive analysis was to use the metabolite concentration values to predict whether a patient would have a positive/negative MRI. For this part, the three patients with unknown findings were removed from the analysis and data from 93 patients were analyzed. All the analyses were performed in R 3.6.1 and for the SVM model the package *e1071* was used.

Two machine learning approaches were evaluated for prediction. Firstly, an SVM model with a radial basis function kernel was applied [52]. For each run the parameters of the SVM model were optimized based on a grid search of the parameter space, based on cross-validation on the testing set. Secondly, a logistic regression model was built as described previously [18]. For each algorithm, 100 models were built, randomly selecting at each run 70% of the observations as training set and 30% of the observations as testing set. For both the SVM and the logistic regression models, sensitivity, specificity, and ROC curve with AUC were calculated. All models were controlled for age and sex. For the best model, the addition of the protein biomarkers to the model was evaluated in order to examine if they can provide a meaningful improvement to the accuracy of the model. Only 71 patients had the full panel of blood proteins available, which were included in this part of the analysis. Finally, since there were 26 patients with negative CT but positive sMRI, we investigated how the predictive models perform on these patients alone.

## 6. Conclusions

Taken together, this study adds further evidence that circulating metabolites may be useful biomarkers in TBI. Here we showed that specific metabolites associate with the specific brain structural changes following TBI, as acquired from sMRI, both grey and white matter injury. Specific metabolites were also found useful in discrimination of positive and negative sMRI findings in a patient cohort consisting mainly of patients with mild TBIs. Combination of this model with a specific protein biomarker, NF-L, further improved the discrimination between patients with sMRI positive and negative findings. Studies in larger cohorts, such as in CENTER-TBI [28,53], are needed in order to validate, further refine, and evaluate the diagnostic utility of the models presented here.

## Figures and Tables

**Table 1:** Demographic characteristics of the study population, including injury classification of MRI findings. SD: standard deviation.

Mean Age (SD)	48.9 (18.9)
Sex	63 males / 33 females
Pre-hospital GCS (SD)	13.5 (3.1)
mTBI	79
moTBI	10
sTBI	7
Injury classification of MRI findings	
Code: 0	26
Code: 1	5
Code: 1,2,3,9	1
Code: 1,3	7
Code: 1,3,4	1
Code: 1,3,5	1
Code: 1,3,5,6,8	2
Code: 1,3,5,6,9	1
Code: 1,3,5,9	1
Code: 1,3,6,9	1
Code: 1,7	1
Code: 1,9	4
Code: 3	3
Code: 3,5,6	1
Code: 3,5,9	2
Code: 3,6,8	1
Code: 3,7,9	1
Code: 3,8,9	1
Code: 3,9	7
Code: 4	1
Code: 4,9	2
Code: 6,8,9	1
Code: 9	22
Code: Unknown	3

**Table 2:** Summary of metabolite clusters.

Cluster No.	n Metabolites	Summary	Examples
1	117	Sugar intermediates, keto acids	d-Mannose, d-galactose, *myo*-inositol, hydroxyisovaleric acid
2	35	Tricarboxylic acid cycle (TCA) intermediates	Lactic acid, pyruvic acid
3	59	Sugar intermediates	Erythrose, gluconic acid, ribonic acid
4	35	Fatty acids	Arachidonic acid
5	24	Mostly unknowns	Glycerid acid
6	51	Fatty acids and intermediates	Oleic acid, stearic acid, adipic acid
7	75	Amino acids, microbial metabolites, sugar intermediates	Glycine, tryptophan, indole-3-propionic acid, erythronic acid
8	55	Amino acids	Leucine, valine, isoleucine, serine, phenylalanine, ornithine

**Table 3:** Relative levels of the important metabolites within the two MRI findings groups. SD, standard deviation.

	Metabolites	MRI Positive		MRI Negative		*p*-values
ID	Name	Mean	SD	Mean	SD	
19	Threonine	6.19	0.72	6.59	0.71	0.017
21	Serine	4.99	0.95	5.47	0.80	0.017
22	Isoleucine	5.79	0.74	6.16	0.71	0.033
25	Glycine	8.16	0.29	8.33	0.38	0.043
49	Erythronic acid	6.61	0.81	7.24	0.32	0.0000004*
53	*Myo*-inositol	7.80	0.40	7.56	0.32	0.003
149	Unknown alcohol	3.94	1.28	4.59	0.79	0.004
188	Unknown sugar derivative	2.64	1.07	3.23	0.90	0.023
192	Hexonic acid	3.16	0.70	2.80	0.78	0.046
312	Pyroglutamic acid	3.61	2.04	4.59	2.11	0.047
380	Unknown carboxylic acid	2.18	1.22	1.49	1.27	0.008
1321	1,4-Benzenedicarboxylic acid	0.18	1.09	0.781	1.13	0.024

* False Discovery Rate < 0.05.

**Table 4:** Injury classification of the MRI findings.

MRI Findings Classification
0 = normal
1 = contusion
2 = EDH
3 = acute SDH
4 = chronic SDH
5 = tSAH
6 = ICH
7 = punctate hemorrhage
8 = diffuse oedema
9 = diffuse axonal injury/white matter damage
